# Antidiabetic activity and phytochemical screening of extracts of the leaves of *Ajuga remota* Benth on alloxan-induced diabetic mice

**DOI:** 10.1186/s12906-017-1757-5

**Published:** 2017-05-02

**Authors:** Tadesse Bekele Tafesse, Ariaya Hymete, Yalemtsehay Mekonnen, Mekuria Tadesse

**Affiliations:** 1 School of Pharmacy, College of Health & Medical Sciences, Haramaya University, Harar, Ethiopia; 2 School of Pharmacy, College of Health Sciences, Addis Ababa University, Addis Ababa, Ethiopia; 3 Department of Biology, College of Natural Sciences, Addis Ababa University, Addis Ababa, Ethiopia; 4 Ethiopian Institute of Agricultural Research, Addis Ababa, Ethiopia

**Keywords:** *Ajuga Remota*, Antidiabetic activity, Blood glucose level, Diabetes mellitus, Glibenclamide, Phytochemical screening

## Abstract

**Background:**

*Ajuga remota Benth* is traditionally used in Ethiopia for the management of diabetes mellitus. Since this claim has not been investigated scientifically, the aim of this study was to evaluate the antidiabetic effect and phytochemical screening of the aqueous and 70% ethanol extracts on alloxan-induced diabetic mice.

**Methods:**

After acute toxicity test, the Swiss albino mice were induced with alloxan to get experimental diabetes animals. The fasting mean blood glucose level before and after treatment for two weeks in normal, diabetic untreated and diabetic mice treated with aqueous and 70% ethanol extracts were performed. Data were statistically evaluated by using Statistical Package for the Social Sciences software version 20. *P*-value <0.05 was considered statistically significant.

**Results:**

The medium lethal doses (LD_50_) of both extracts were higher than 5000 mg/kg, indicating the extracts are not toxic under the observable condition. Aqueous extracts of *A*.*remota* (300 mg/kg and 500 mg/kg body weight) reduced elevated blood glucose levels by 27.83 ± 2.96% and 38.98 ± 0.67% (*P* < 0.0001), respectively while the 70% ethanol extract caused a reduction of 27.94 ± 1.92% (300 mg/kg) & 28.26 ± 1.82% (500 mg/kg). Treatment with the antidiabetic drug, Glibenclamide (10 mg/kg body weight) lowered blood glucose level by 51.06% (*p* < 0.05). Phytochemical screening of both extracts indicated the presence of phenolic compounds, flavonoids, saponins, tannins, and steroids, which might contribute to the antidiabetic activity. The extracts, however, did not contain alkaloids and anthraquinones.

**Conclusion:**

The aqueous extract (500 mg/kg) showed the highest percentage reduction in blood glucose levels and the ability of *A. remota* extracts in reducing blood glucose levels presumably due to the presence of antioxidant constituents such as flavonoids. The effect of the extract supported the traditional claim of the plant.

## Background

Diabetes mellitus, one of the major public health problems worldwide, is a metabolic disorder of multiple etiologies distinguished by a failure of glucose homeostasis with disturbances of carbohydrate, fat and protein metabolism as a result of defects in insulin secretion and/or insulin action [1, 2]. According to International Diabetes Federation (IDF) report, elevated blood glucose is the third uppermost risk factor for premature mortality, following high blood pressure and tobacco use globally [2].

Cardiovascular diseases, neuropathy, nephropathy, and retinopathy are among the major risks that are associated with diabetes. These chronic complications may lead to hardening and narrowing of arteries (atherosclerosis) that could advance to stroke, coronary heart disease, and other blood vessel diseases, nerve damage, kidney failure, and blindness with time [3].

In 2015, according to IDF report, 415 million (8.8%) adults (aged 20–79) worldwide were estimated to have diabetes; this number is expected to rise to 642 million (10.4%) by 2040 or one adult in ten people. An estimated 14.2 million adults aged 20–79 had diabetes in the Africa Region that represents a regional prevalence of 3.2% (2.1–6.7%) in 2015, which can be projected to 3.7%(2.6–7.3%; 34.2 million) by 2040. South Africa (2.3 million), Democratic Republic of Congo (1.8 million), Nigeria (1.6 million) and Ethiopia (1.3 million) are among the highly populated African countries containing the highest number of people living with diabetes [2].

In Ethiopia, even though the occurrence of diabetes has not been made nationally, hospital-based studies indicate that its prevalence has increased from 3.32% in 2012 [4] to 3.4% in 2015 and the number of diabetic cases is expected to increase to 10.6 million by 2040 [2].

Diabetes mellitus can be managed by diet, physical exercise, and modern drugs (insulin and/or oral hypoglycemic drugs such as sulfonylureas and biguanides) [5]. Different extracts from medicinal plants have also been used traditionally to manage diabetes globally, and these are considered as relatively inexpensive, less toxic and with relatively little or no side effects [6]. There are also medicinal plants that contain some toxic constituents such as the cytotoxic anti-cancer plant-derived drugs, digitalis; however, the side effects of the phytotherapeutic agents are less common compared with synthetic drugs [7]. Management of diabetes without any side effect is still a challenge and the available modern antidiabetic agents produce serious side effects such as hypoglycemia (Sulphonylureas), lactic acidosis and folate and B12 malabsorption (Metformin), gastrointestinal symptom (Acarbose), weight gain (Sulphonylureas and Thiazolidinediones), and edema (Thiazolidinediones) [8]. Hence, the search for safer and more effective hypoglycemic agents has continued.

Globally, medicinal plants have been used as a source of medicine and 80–85% of populations rely on these medicinal plants using the extracts or their active components as traditional medicine to meet their primary health care needs [9, 10]. A number of active components were isolated from medicinal plants for direct use as drugs, or act as a lead compound or pharmacological agents. Metformin, for example, is an oral hypoglycemic agent isolated from medicinal plant *Galega officinalis* that was used historically in medieval Europe for the treatment of diabetes [11–13].

*Ajuga remota Benth* (synonyms: *Ajuga integrifolia Buch.-Ham, Ajuga bracteosa Wall ex Benth*.) [14], is a shrub in the Lamiaceae family that grows widely in East Africa, at an altitude of 1500–3400 m above sea level in Saudi Arabia, Yemen and Afghanistan to East Asia [15, 16]. In the Ethiopian traditional health system, *A.remota* is commonly used for the treatment of various diseases including diabetes, malaria, toothache, skin disease, high blood pressure, stomach pain, pneumonia, liver problem and swelling of legs. Due to the bitter taste of the extract, sometimes honey is added to the preparation so as to make it palatable and for longer period storage for later use. In Ethiopia, the vernacular names for *A.remota* include ‘Harmagussa’ (Ormigna), ‘Akorarach’ (Amharic); ‘Etse-Libawit’ (Ge’ez), ‘Akembiye’ (Guragegna), and ‘Tale’ (Welaytigna) [15–20].

The plants of the genus *Ajuga* have been assessed for different activities such as antiviral activity against Human Immunodeficiency Virus type 1 (HIV-1) and Type 2 (HIV-2) [21], antipyretic [22], diuretic [23], anti-inflammatory, antidepressant, anticoagulant [24], analgesic [24, 25], antiarthritic [26], antifeedant, antifungal, antihypertensive, insecticidal [18, 27, 28], antimicrobial [29], antioxidant [24, 29, 30], hypoglycemic [31–35], antinociceptive [36], hypolipidemic [37], antimycobacterial [38], and antimalarial/antiplasmodial activities [18, 19, 27, 28, 39, 40].

To verify the traditional uses of the plant *Ajuga* genus, various in vitro and in vivo studies have been conducted on different extracts of the species of the genus *Ajuga* using animal models, such as mice or rats; as their genetic, biological and behavior characteristics closely resemble human beings. *A.remota*, *A.bracteosa*, *A.integrifolia*, and *A.iva* are some of the species of the genus *Ajuga* that are ecologically related and were evaluated for various pharmacological applications.

Different extracts of *A.bracteosa wall ex. Benth* was evaluated for its analgesic effect using Swiss albino mice with acetic acid-induced writhing and tail immersion test. A dose-dependent analgesic effect was observed at 200 and 400 mg/kg from the water and chloroform extracts [25]. In another study that used *A.bracteosa Wall ex Benth*, its antiarthritic effect was evaluated in albino rats using a 70% ethanolic extract and dose-dependent activities with a better effect than that of aspirin was observed after six h treatment on acute non-immunological arthritis and complete Freund’s adjuvant (CFA)-induced chronic immunological arthritis. The antiarthritic effect was reported to be due to the presence of the active constituents’ ajugarin I, lupulin A, withaferin A, reptoside and 6-deoxyharpagide that were isolated from the plant [26].

In Taiwan, in vitro and in vivo hypoglycemic effect was evaluated on the 70% ethanol extracts of five *Ajuga* species namely; *A.decumbens*, *A.nipponensis*, *A.pygmaea*, *A.taiwanensis* and *A.dictyocarpa* on streptozotocin-induced diabetic mice. Among them, *A.nipponensis showed* a superior effect in α-glucosidase inhibition (28.62 ± 1.56%) and glucose uptake (54.15 ± 2.56%) and somewhat postprandial blood glucose levels reduction. It also contains the highest content of flavonoids and ecdysterone as compared with the other species [32].

A phytoecdysteroids rich aqueous extract from *A.iva* on alloxan-induced diabetic rats significantly (*p* < 0.01) decreased the level of blood glucose level and increased hepatic glycogen levels [34]. In another study, the aqueous extract of whole parts of *A.iva* was examined for hypolipidemic and hypoglycemic effect on streptozotocin-induced diabetic rats and a significant reduction in plasma cholesterol and triglyceride levels were observed by 35% (*P* < 0.01) and 13% (*P* < 0.05) [35] and 44% (*P* < 0.01) and 30% (*P* < 0.01) [37], respectively while the plasma levels of glucose was reduced by 24% (*P* < 0.05) [35].

*A.remota* and the other species of the genus *Ajuga* have a number of common phytochemical compounds. Although ample ethnobotanical, in vitro and in vivo evidence exists for the use of *Ajuga* species in the management of diabetes, the claim of *A.remota* has not been substantiated scientifically. Therefore, the aim of this study was to find out the scientific basis of the use *A.remota* in the management of diabetes used by traditional practitioners using aqueous and 70% ethanol extracts on alloxan-induced diabetic mice.

## Methods

### Collection of plant material

The leaves of *Ajuga remota* Benth were collected from Lebu, a few kilometers Southwest of Addis Ababa, Ethiopia in January 2008. Taxonomic identification was made by Mr. Melaku Wondafrash at the National Herbarium, College of Natural Sciences, Addis Ababa University and a voucher specimen (Voucher Specimen number T001) was preserved.

### Chemicals and instruments

Alloxan monohydrate (Sigma Chemical Company, USA) was used to induce diabetes in mice and Glibenclamide (Hoechst Pharmaceuticals, Mumbai) was used as a standard hypoglycemic drug. Ethanol (BDH Ltd., England) and distilled water were used for extraction of the plant materials. ACCU CHEK Performa Glucometer (Roche Diagnostics India Pvt. Ltd., India) was used to measure the blood glucose level. For evaporating the solvents, BUCHI Rotavapour R-200, Switzerland and Lyophilizer (freeze dryer) (type: Heto power dry LL3000 Wag tech) was used. The following chemicals were used for phytochemical screening test: Chloroform and Ethyl acetate (ACS, Merck); Hydrochloric acid, Ferric sulphate, Lead acetate and Potassium ferrocyanide (BDH Ltd., England); Petroleum ether 60-80 °C (Labmerk Chemicals LTD India); Sulphuric acid (Farm Italia Carrloerba, Italy); Acetic anhydride and Methanol HPLC grade (Techno Pharmchem, Bahadurgarm, India); n-Hexane (Rathburn Chemicals Ltd., England); Acetonitrile (Sigma Aldrich, Germany) and Ferric chloride (FISHER Scientific Company, USA). All the chemicals were of analytical grades.

### Experimental animals

Adult male Swiss albino mice bred in the animal house of Ethiopian Health and Nutrition Research Institute, Addis Ababa, Ethiopia, with weights ranging from 24 to 35 g and eight weeks of age were used for the experiment. The animals were kept in cages made of polypropylene (5 mice per cage randomly) at 23 ± 2 °C with 12 h/12 h light/dark cycle [41–44]. Standard pellet and water were allowed to the animals throughout the experiment, except the fasting period. The care and handling of animals were in accordance with internationally accepted ethical guidelines for use of laboratory animals [44] and the study protocol was approved by the School of Pharmacy Ethics Committee, Addis Ababa University, Ethiopia.

### Extraction of plant material

The leaves of *A.remota* were air-dried for one week under the shade at room temperature at Essential Oils Research Center laboratory, Addis Ababa. The dried plant material was manually powdered finely and used for extraction.

### 70% ethanol extract

One hundred gram of the dried and powdered leaves of *A.remota* was kept in a thimble and extracted with 70% ethanol in a Soxhlet extractor. The extraction process was continued until the color of the final drop of the extract became colorless. Then, ethanol was removed from the extract using a rotavapor (BUCHI Rotavapor R-200, Switzerland) at 60 °C and the remaining 30% water was removed using Lyophilizer (freeze dryer) (type: Heto power dry LL3000 Wag tech). The resulting dry hydroalcoholic extract has a percentage yield of 10.95% (*w*/w). The extract was kept in a refrigerator until used for the experiment.

### Aqueous extract

Twenty gram of the dried and powdered leaves of *A.remota* was added to 100 mL hot distilled water (60^o^c), mixed thoroughly and heated for 20–30 min. on a water bath with continuous stirring as traditionally done and cooled to room temperature. The decoction obtained was filtered under suction and Lyophilizer (freeze dryer) (type: Heto power dry LL3000 Wag tech) was used to dry the aqueous extract. The dried aqueous extract has 13.5% (*w*/w) percentage yield. The freeze-dried aqueous extract was kept in a refrigerator until used for the experiment and then the dried plant extract was reconstituted with distilled water for oral administration.

### Acute toxicity test

Acute toxicity test were done on both plant extracts after the animals had been fasted overnight while only taking water [45]. The weight of each mouse was recorded before administering the extract. Randomly the animals were divided into a control and three treatment groups (separately for both extracts), each group consisting of five mice. The control group received only the vehicle (1% Tween 80) and each treatment group received orally the 70% ethanol and aqueous extracts of *A.remota* in a dose of 1000, 2000 and 5000 mg/kg [46]. Animals were kept under close observation for explicit toxicities and/or behavioral changes like restlessness, tremor, diarrhea, sluggishness, loss of weight, and paralysis at regular intervals for the first four h after administering the extract [47], and then they were observed daily for two weeks for any change in general behavior and/or other physical activities. Food was available after four h of administration of the extracts.

### Induction of experimental diabetes

Male Swiss albino mice were fasted overnight (12–14 h) and their weight and fasting blood glucose level recorded with a glucometer and then made diabetic by a single intraperitoneal injection (a volume of 1 mL/kg) of freshly prepared alloxan monohydrate solution (20 mg/kg body weight). Alloxan was prepared by weighing according to individual animal weight and solubilized with 0.5 mL sodium citrate at pH 4.5 before injection. Food and water were presented to the animals 30 min. after administration of alloxan [48, 49]. After 48 h of alloxan injection, plasma blood glucose level of each animal was determined by taking the blood from the tail and animals with a fasting blood glucose level above 200 mg/dL [50, 51] were included in the study.

### Experimental design

The animals were divided into seven groups for the evaluation of fasting blood glucose level and oral glucose tolerance test with five animals in each group. They were treated with the plant extracts two days after alloxan injection excluding the diabetic control groups. Blood samples were drawn for measuring blood glucose levels from each group on day 1, 7 and 14 during the study period [43]. Changes in body weight were also recorded. Groups 1 and 2 served as normal and diabetic controls (receiving only the vehicle, i.e. 1% Tween 80), respectively. Group 3 received standard drug (glibenclamide, 10 mg/kg per day orally) [45]. Groups 4 & 5 received the 70% ethanol extract at a dose of 300 & 500 mg/kg, respectively and groups 6 & 7 received aqueous extract at a dose of 300 & 500 mg/kg, respectively daily in one mL aqueous solution using oral gavage for two weeks.

### Oral glucose tolerance test

After two weeks of treatment with the plant extracts, the animals were made to fast for 12–14 h but had free access of water and their fasting blood glucose level was measured four times. Glucose solution (2 g/kg of body weight) was administered orally in a volume of 1 mL/kg. Blood samples were collected at the time interval of 30, 60 and 120 min. after administration of glucose [41].

### Phytochemical screening

Preliminary phytochemical screening of the plant extracts was carried out using standard procedures [52], to check for the presence or absence of secondary metabolites such as alkaloids, steroidal compounds, phenolic compounds, flavonoids, saponins, tannins and anthraquinones.

### Statistical analysis

Data are expressed as a mean ± standard deviation. Differences among treatment group means were assessed by two-way analysis of variance (ANOVA) and group means were considered to be significantly different at *P* < 0.05. Data were statistically evaluated using Statistical Package for the Social Sciences (SPSS) version 20.0 software. Bar and line charts were drawn using Excel 2007 software.

## Results and discussion

### Acute toxicity test

The acute toxicity study showed that the administration of graded doses of both the aqueous and 70% ethanol extracts of *A.remota* did not generate any observable signs of toxicity up to the dose of 5000 mg/kg, which is consistent with Debela et al., 2005 [22] and Hailu and Engidawork, 2014 reports [23]. This was confirmed by the absence of significant changes in behaviours such as alertness, motor activity, weight loss, sluggishness, paralysis, breathing, restlessness, diarrhoea, convulsions, and coma. In addition, no death was observed for two weeks and they were physically active. The result proves that the plant extracts had no observable adverse effect at the doses tested; implying that the medium lethal dose (LD_50_) is greater than 5000 mg/kg body weight in mice. Since its actual median lethal dose (LD50) is greater than 5000 mg/Kg, extract of *A.remota* is non-toxic [53].

### Effect of *A. remota* leaves extract on fasting blood glucose level

**Fig. 1:**
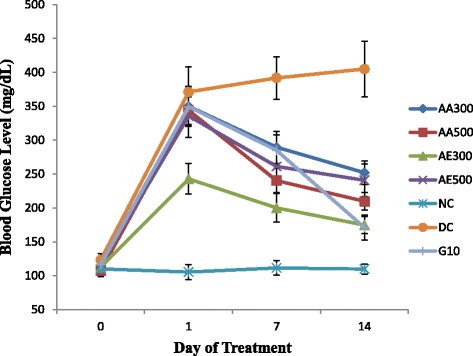
Effect of extracts of *A.remota* on fasting blood glucose level in normal control and alloxan-induced diabetes mice. AA300: Aqueous Extract of *A.remota* (300 mg/kg per day); AA500: Aqueous Extract of *A.remota* (500 mg/kg per day); AE300: 70% Ethanol Extract of *A.remota* (300 mg/kg per day); AE500: 70% Ethanol Extract *A.remota* (500 mg/kg per day), NC: Normal Control; DC: Diabetic Control; G10: Glibenclamide (10 mg/kg per day). Data was expressed as mean ± SD (*n* = 5)

Alloxan-induced diabetic mice were treated with aqueous and 70% ethanol extracts of *A.remota*, once a day orally, for 14 days. The effect of different doses of the extracts of *A.remota* on fasting blood glucose level is presented in Fig. 1. The present study was intended to examine the antidiabetic effects of the extracts of *A. remota* leaves. The dose of *A. remota* (300 and 500 mg/kg body weight) was selected based on previous studies in the *Ajuga* species [32, 36].

Alloxan monohydrate has been used to induce diabetes mellitus in experimental mice [48, 49]. A single intraperitoneal administration of 20 mg/kg body weight alloxan monohydrate solution induced effectively diabetes mellitus in mice. This was confirmed by elevated level of fasting blood glucose that can be obtained from the tail of the mice after 48 h of injection.

Alloxan brings diabetes through selective destruction of insulin secreting pancreatic β-cells due to its accumulation through the glucose transporter 2 (GLUT2) and hence, minimize the glucose uptake by peripheral tissues. It is known that alloxan induces free radical formation by redox reactions that cause tissue injury and make ß-cells to degranulate and consequently degenerate [48, 54].

As expected in the diabetic control there was a 9.1% ± 1.5 increases in mean blood glucose level and significant difference (*p* < 0.0001) with the normal control mice. The blood glucose level of diabetic mice was estimated before and after 1st, 7th and 14th days of treatment. Both the aqueous and 70% ethanol *A. remota* extract treatment groups show a statistically significant difference with normal and diabetic control mice with *p* < 0.0001. There is also a statistical significant difference between each dose of aqueous extracts (*P* < 0.005) and the 70% ethanol extract (300 mg/kg) (*P* < 0.0001). However, there were no significant differences (*P* > 0.05) among the 70% ethanol extract with 500 mg/kg (AE500) and both aqueous extracts and Glibenclamide.

The average percentage of decrease in blood glucose levels (Table 1) showed an increase in the percentage with a relative increasing dose administration of *A. remota* extracts. However, the aqueous extract of *A. remota* with dose of 500 mg/kg body weight had a greater percentage decrease (38.98 ± 0.67) than any of the extracts after 14 days of treatment administration. Glibenclamide (10 mg/kg body weight) treated diabetic mice showed a 51.10 ± 2.95 percentage reductions as positive control.

**Table 1:** Average Percentage Decrease of Blood Glucose Level (% DBGL)

Group of Treatments	DBGL (%)
Diabetic + Aqueous *A. remota* Extract (300 mg/kg body weight)	27.83 ± 2.96
Diabetic + Aqueous *A. remota* Extract (500 mg/kg body weight)	38.98 ± 0.67
Diabetic +70% Ethanol *A. remota* Extract (300 mg/kg body weight)	27.94 ± 1.92
Diabetic +70% Ethanol *A. remota* Extract (500 mg/kg body weight)	28.26 ± 1.82
Diabetic + Glibenclamide (10 mg/kg body weight)	51.10 ± 2.95

The extracts of *A. remota* leaves reduced elevated fasting blood glucose level. The mechanism of antidiabetic effects of the extracts of *A. remota* leaves might be due to presence of well-known antioxidant phytochemicals like flavonoids, polyphenols, and tannins, which acts as a free radical scavengers [54, 55]. The presumed mechanism of action of these antioxidants was because of an insulin mimetic effect on the peripheral tissues by either stimulation of regeneration process or release of pancreatic secretion of insulin from existing β-cells. On top of this mechanism; there are also other mechanisms that play a great role in the reduction of blood glucose levels as potential antidiabetic plants. Increasing the speed of the release of glucose from the circulation by accelerating filtration and renal excretion and increasing the release of glucose through enhanced metabolism or integrate into fat deposits, a process relating to the pancreas to produce insulin are among the others [54].

A significant number of compounds have been isolated from various species of the *Ajuga* herb including; sterols (ajugalactone, β-sitosterol, γ-sitosterol, stigmasterol,), phenolic components, arabinose, cerotic acid, ecdysterone, phytoecdysteroids (phytoecdysteroid, Cyasterone, ajugalactone, ajugasterone A-C), flavonol glycosides, triterpenoid (ergosterol-5,8-endoperoxide), iridoid glycoside (8-O-acetylharpagide, 6,8-diacetylharpagide, Ajureptoside, 8-acetylherpagide, Herpagide,), neoclerodane-diterpenes and diterpenes (ajugarins I, II, IV and V) [16, 19, 28, 38, 56–58].

The result of this research supported the traditional use of the plant extract for the management of diabetes as claimed by traditional practitioners in Ethiopia. However, we are not certain about the mechanism of the antidiabetic action of the extracts. Therefore, further investigation needs to be performed.

### Oral glucose tolerance test

**Fig. 2:**
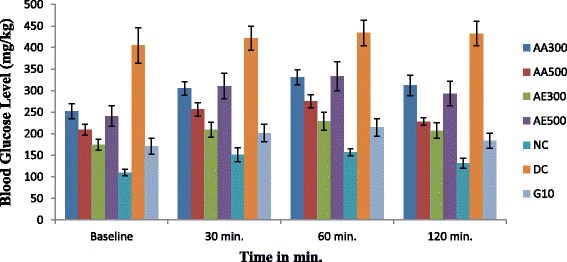
Glucose tolerance test of *A.remota* extracts on alloxan-induced diabetic mice. AA300: Aqueous Extract of *A.remota* (300 mg/kg per day); AA500: Aqueous Extract of *A.remota* (500 mg/kg per day); AE300: 70% Ethanol Extract of *A.remota* (300 mg/kg per day); AE500: 70% Ethanol Extract *A.remota* (500 mg/kg per day), NC: Normal Control; DC: Diabetic Control; G10: Glibenclamide (10 mg/kg per day). Data was expressed as mean ± SD (*n* = 5)

The mean blood glucose levels of normal (negative control), diabetic mice untreated (positive control) and diabetic mice treated with *A.remota* extracts that were subjected to glucose tolerance test after two weeks is presented in Fig. 2. The animals in each group (*n* = 5) fasted 12–14 h and then the fasting mean blood glucose level was evaluated after oral administration of glucose (2 g/kg body weight) as a baseline. The mean blood glucose level in the normal control mice rise to a peak value after 60 min glucose load and decreased to near normal level after 120 min. In diabetic control mice, however, the value increased to a peak after 60 min of glucose load and remained high over the next 60 min, which is expected.

The animals that were treated with the extracts showed a reduction in the mean blood glucose levels after 60 min of glucose loading. At 60 min, the blood glucose level reached the maximum in both the aqueous and 70% ethanol extracts treated animals and then significant reduction was observed after 120 min of glucose administration. The aqueous extract at a dose of 500 mg/kg body weight showed a better reduction with 17.21% from the peak blood glucose level.

### Phytochemical screening

Preliminary phytochemical screening was carried out using color forming and precipitating chemical reagents for detecting plant constituents from their extracts. The results obtained from the tests were summarized in Table 2, indicating the presence of flavonoids, tannins, saponins, phenolic compounds and steroids. This result was consistent with previous reports [16, 19, 28]. The extracts didn’t contain alkaloids and anthraquinones. Epicatechin (flavonoids), catechin (tannin) and vindoline (an alkaloid) were some of the documented compounds that were isolated from the plant with a potential to decrease the blood glucose level [13, 42, 45]. Thus, the significant antidiabetic effect of the extracts of *A.remota* could be due to the presence of the abovementioned components in the extracts, which could act synergistically and/or independently to enhance the activity of glycolytic enzymes.

**Table 2:** Phytochemical screening of the aqueous extract of *A. remota*

Tests	Reagents	Inferences
Alkaloids	Dragendorff’s	−
	Mayer’s	−
Anthraquinones	Test for free anthraquinones	−
	Test for o-anthraquinone glycosides	−
Flavonoids	10% Lead acetate	+
	Sodium hydroxide	+
	Ethyl acetate	+
Phenolic compounds	Ferric chloride and potassium ferrocyanide	+
Saponins	Froth test	+
Steroidal compounds	Acetic anhydride and conc. Sulfuric acid	+
	Chloroform and conc. Sulfuric acid	+
Tannins	Ferric chloride	+
	Aqueous hydrochloric acid	+
	Formaldehyde	+
	Modified iron complex	+

Positive and negative symbol indicates the presence and absence of plant constituents, respectively.

## Conclusion

Oral administration of the extracts in doses from 1000 to 5000 mg/kg/day did not produce any significant changes in behaviors, indicating that the extracts are not toxic under the observable condition in mice. The leaves of *A.remota* extracts had flavonoids, tannins, saponins, phenolic compounds and steroids, where some are considered as bioactive constituents in the management of diabetes. The *A. remota* extracts with each dose of 300 and 500 mg/kg body weight had antidiabetic effects on alloxan-induced Swiss albino mice. Percentage reduction in blood glucose levels of *A. remota* aqueous and 70% ethanol extract with dose of 300 and 500 mg/kg body weight each are 27.83 ± 2.96 and 38.98 ± 0.67 and 27.94 ± 1.92 and 28.26 ± 1.82, respectively. Hence, the chemical constituents of the plant extract might help in preventing diabetic complications and may serve as an alternative in the present armamentarium of antidiabetic drugs. Further study to substantiate the use of the plant as antidiabetic is recommended.

## Abbreviations

AA300: Aqueous extract of *Ajuga remota* 300 mg/kg/day body weight
AA500: Aqueous extract of *Ajuga remota* 500 mg/kg/day body weight
AE300: Ethanol extract (70%) of *Ajuga remota* 300 mg/kg/day body weight
AE500: Ethanol extract (70%) of *Ajuga remota* 500 mg/kg/day body weight
DC: Diabetic Control
G10: Glibenclamide 10 mg/kg/day body weight
HIV-1/2: Human Immunodeficiency Virus type 1 and type 2
LD_50_: Medium Lethal Dose
NC: Normal control
SD: Standard Deviation
